# Impact of implementing multisource feedback on behaviors of young doctors

**DOI:** 10.12669/pjms.37.7.4155

**Published:** 2021

**Authors:** Ch. Nasir Ahmad, Ahsan Sethi, Rehan Ahmed Khan

**Affiliations:** 1 Dr. Ch Nasir Ahmad, MBBS, FCPS, FICO, Fellowship in vitreo-retina, MME. Department of Ophthalmology Unit-II, King Edward Medical University, Lahore, Pakistan; 2 Dr. Ahsan Sethi, BDS MPH MMEd FHEA MAcadMEd FDTFEd PhD. Department of Public Health, College of Health Sciences, QU Health, Qatar University, Doha, Qatar; 3 Prof. Dr. Rehan Ahmed Khan, MBBS, FCPS, FRCS, JMHPE, MSc-HPE, MHPE. Islamic International Medical College, Riphah International University, Islamabad, Pakistan

**Keywords:** Multisource Feedback, Ophthalmology, Pakistan, Qualitative, Research, Surgeons, Workplace based assessment

## Abstract

**Objectives:**

Multisource feedback (MSF) is a workplace-based assessment tool that offers 360-degree evaluation of the trainee doctor. Little is known about its receptiveness among stakeholders in Pakistan. This study explores house officers’ perceptions regarding MSF since its implementation in Eye Unit-II, Institute of Ophthalmology, King Edward Medical University/ Mayo Hospital, Lahore.

**Methods:**

A qualitative case study was conducted from July 2019 to February 2020 in Eye Unit II. A purposive (maximum variation) sample of 12 house surgeons was taken. Two focus group discussions were conducted. Data were transcribed and analyzed thematically.

**Results:**

The study identified the impact of MSF on house surgeons. Most participants reported positive experiences. The feedback they received increased their motivation, management skills and team working. A number of factors affecting the receptiveness of MSF were also identified which mainly included characteristics of raters and emotional response to MSF.

**Conclusion:**

Multisource Feedback is a useful tool for feedback that impacts the young doctors in many ways. It contributes to increasing their sense of responsibility, management skills and self-directed learning. The improvement in individual abilities and teamwork also helped in improving patient care.

## INTRODUCTION

Multisource feedback (MSF) is a workplace-based assessment tool to help provide feedback on the non-technical skills and attitude of professionals. Multisource feedback has four basic components “senior, junior, peer colleague, and self- assessment”.^1^

MSF is being used as a tool for assessment of professional development of health professionals in all over the world^2^-^4^ Giving appropriate feedback is an important component of MSF which allows the health care professionals to reflect their performance and improvement.^5^ The basic catalyst for change is negative or discrepant feedback.^6^ This change can be both positive and negative, however, despite a positive shift in the MSF, negative responses may also occur as a result of negative feedback.^7^,^8^

The skills and attitude of the health professionals are frequently questioned in our local perspective and across the globe.^9^ This may be due to a lack of useful feedback from the seniors and colleagues about their clinical practice.

Surprisingly there are few published articles that explore the impact of MSF and the factors that influence the effectiveness of MSF on physician behavior.^6^ In Pakistan only three research articles are published on MSF,^10^-^12^ all of these discuss the implementation of MSF on doctors and the resulting behavior change however none of these explore reactions to MSF and its likely impact on behavior.

The purpose of this study was to explore the impact of multi-source feedback on the behavior of young doctors and to identify the factors affecting the receptiveness of MSF on house officers.

## METHODS

This Qualitative case study was conducted among house officers working in Mayo hospital, Lahore, Pakistan, from July 2019 to February 2020. The project was reviewed and approved by the Ethical Review Board of The University of Lahore (Ref: ERC/12/19/03 dated on December 30, 2019).

### Study Setting

MSF is being carried out in Eye Unit II, Institute of Ophthalmology, Mayo Hospital, Lahore as a part of performance assessment at the end of three months rotation of house officers. MSF was conducted with mini-Peer Assessment Tool(mini-PAT). Facilitators were provided training to conduct MSF. Results of MSF were disclosed to the participants while maintaining confidentially.

### Data Collection

A purposive (maximum variation) sample of 12 house officers were selected. A focus group discussion guide was developed based on AMEE Guide no.91 which included questions that explored the impact of MSF on behavior and identified the factors affecting the receptiveness of MSF. Two focus groups were conducted for the purpose of data collection with six respondents in each FGD. Research questions were identified from pilot study. Open-ended questions that reflected our objectives were selected.

### Data Analysis

Data analysis was done based on the thematic analysis framework formulated by Braun V & Clarke V.^13^ Kirkpatrick model^14^ was used as a conceptual framework. Open ended questions were asked from each focus group which was both video and audio-taped for validity. Transcripts were defined from the qualitative data; transcripts were reviewed by all researchers to develop a coding framework. Data was analyzed again and themes were generated. Data was reviewed and refined again in light of themes. Those themes were defined and named and finally on the basis of this final report was written. Further analysis was done based on constant comparative method.

## RESULTS

The participants (House Officers) involved in the study had 1:1 male to female ratio, similar age groups and academic qualifications. Ten house officers out of 12, were graduates of batch 2019, only two were from previous batches. Table-I provides the details of study participants.

**Table I:** Demographic characteristics.

*Demographic characteristics*	*Frequency*	*Percentage*
***Gender***		
Male	6	50%
Female	6	50%
***Age groups***		
Mean Age	22.3	
20-22 years:	7	58%
23 years or more	5	42%
***Year of Graduation***		
2019	10	83%
2018-2017	2	17%
***Academic Qualification***		
Basic Qualification MBBS	12	100%
***Gap in previous qualification and enrolling (Months)***		
2 months	10	83%
12 months	2	17%

Data analysis led to the identification of 21 subthemes distributed under three themes. Impact of the MSF was seen through Level 3a of Kirkpatrick model by self-reported changes in participants behavior, details given in Table-II.

**Table II:** Impact of Multi-Source Feedback on Behavior.

*Themes*	*Subthemes*		*Excerpt*
Reactions	Orientation		“It is good for an outline at the start of your career that what skill set you need for your future life in workplace.”
	Increased motivation		“It gives insight about you. It brings passion for improvement and eagerness to learn”
	Identifies strengths and deficiencies		“MSF is a good tool as seniors score you, tell you about your deficiencies and strengths.”
Learning	Professionalism		“I learnt professionalism and that how to maintain my performance especially when my seniors are keeping an eye on me.”
	Improved attitude		“So, I was being rated as below expectation regarding my learning attitude and time management skills. So, I focused my attention to these areas and made improvement.”
Behavior	Increased sense of responsibility		“After MSF I improved myself in punctuality and sense of responsibility.”
	Self-directed learning	Courses	“After MSF I got to know about my deficiency and I took courses in communication skills and anger management and character building so it bought positive changes in me”
		Mentors:	“When I came to know about my deficiencies, I looked towards my senior for guidance. I took mentorship of my professor and followed his footsteps.”
		Peer	“I asked my colleagues about how they handled the issues which I have. I ultimately followed my colleague who was excellent in the fields I was lacking in it helped me to be better in such tasks”
	Management skills		“I improved my management skills and now I go in a very systematic way and it has helped me.”
	Improvement in teamwork		“After MSF I came to know that our team is not supposed to be of doctors only, we should respect all our allied staff and so now I have developed a good working relationship with them”
	Improvements in patient care		“It improved my empathy towards the patients, it polished my clinical skills and my approach towards the patients.”
	Recognition of others contribution		“I was lacking basically in recognition of others contribution in services. So I got to know that I don’t give others the credit of their work. It has helped me a lot to improve this”
	No change		“I was quite furious about my feedback as I was doing the best according to me. So, I did not make any change rather I was taken a back from quite a few things”.

### The impact of MSF on the behavior of doctors

Impact of the MSF was seen through Level 3a Self-reported changes in participants behavior of Kirkpatrick model.^14^ The initial reaction to implementation was positive with majority of participants acknowledging the deficiencies such as lack of reliability, lack in communication skills and lack of time management was repeatedly mentioned. This acknowledgment led to a positive learning behavior through which the participants took part in various activities to improve themselves ultimately resulting in behaviors changes.

The behavior changes inculcated were both positive and negative but a few participants did not change. In majority changes were positive, minority had negative change while one participant did not changes owning to their reasons as mentioned in next heading.

### Enablers and barriers to the receptiveness of the feedback

Factors affecting the receptiveness of feedback were analyzed by gathering data based on level-1 and level-2 of Kirkpatrick model.^14^ Multiple factors were identified which were further grouped as enablers and barriers. Raters characteristics, content of feedback and format of the feedback were identified as potential enablers increasing the receptiveness of feedback. These are summarized in the Table-III

**Table III:** Enablers of the Multi Source Feedback.

*Categories*	*Reference quotations*
Raters characteristics	“The raters should be those individuals that you have an interaction with. Head Nurse has barely spoken to me since I came.”
	“The sincerity of seniors that they actually want me to improve compelled me to improve my skills and behavior.”
	“It was given in a very positive environment and affectionate way making me actually receptive to it.”
	“All the raters must be transparent. It helped me because all the individuals were neutral it gave me a blueprint of myself. ”
Content of feedback	“According to me the confidentiality of the feedback was important as if a person is weak in something you do not go and announce it to all demoralizing him in front of all.”
	“It must be transparent if not, ultimately it will be harmful for him instigating negative behavior”
	“To get the feedback from multiple seniors saying the same things individually independent from each other adds to transparency, quality and credibility making a sharp impact on me.”
	“To get the feedback from multiple seniors saying the same things individually independent from each other adds to transparency, quality and credibility making a sharp impact on me.”
	“Everybody gave me the lowest remarks, however when I asked my other colleagues they did not approve of my feedback.”
	“It should be bilateral i.e., junior doctors can also have concerns regarding some of their seniors.”
Format	“I think Implementing the MSF at earlier Level such as schools, Colleges and medical schools will have a greater impact”
	“The assessment must be at two parts, one at the start and then onwards every three months to know how much improvements have you made.”
	“The format is quite good but it should include orientation session at the start of MSF which should give an outline to the rater about what aspects you will be judged in.”

Similarly, a number of barriers to MSF were highlighted during focus group discussion. These included behavior of raters, human resource, lack of confidentiality, time constraints, compliance. Table-IV provides the summary of these barriers

**Table IV:** Barriers to Receptiveness of Multi Source Feedback.

*Categories*	*Reference quotations*
Behavior of raters	“If raters are biased, they will demoralize the participant and ultimately it will be harmful for him instigating negative behavior”
	“It is a human nature that nobody likes to be judged by other and I think it is a major barrier the second thing is that if your results are a label that marks you then definitely this will be a major barrier”
	“Acceptance of criticism is a key point to success of MSF. Tolerance should be encouraged.”
	“Negative impact was due to some aspects such as favoritism that can affect the feedback process”.
	“If you are judged then you will be treated the same way. That the student is not good and we are not going to work on him. It will be difficult to bring change.”
	“Participants must be willing to change if not then it can be a barrier to bring a positive impact.”
Human resource	“Special people professionals must be appointed for these. If not make shift people will make errors and will become a barrier”
	“The raters must be those individuals who are in direct contact, like head of the staff nurse rarely has an interaction with house officers.”
Lack of confidentiality	“Yes, secrecy is the major point in making the Impact. Once confidentiality will be maintained then it will have a positive impact and it will not be barrier”
Time constraints	“It is a long process it takes time, do our seniors have enough time to give feedback to the juniors?”
Compliance	“Compliance is a major problem, with time both doctors and other people will be less compliant to the feedback”

## DISCUSSION

In this study, the impact of implementing the multisource feedback on behaviors of house officers and factors affecting the receptiveness were reviewed, using Kirkpatrick model as conceptual framework. The house officers reported using feedback provided as a result of MSF process, as a way to bring changes to their behavior. Majority of the participants reported likely improvements in behavior while a few participants also mentioned potential barriers to the receptiveness of MSF, if not used properly.

The strongest evidence of positive impact through this feedback has been seen in studies assessing improvement, including an RCT by Smither et al.^15^ and a prospective longitudinal cohort done by Violato et al.^16^ Several other studies have also reported a positive response to MSF; participants were seen either considering or had initiated changes following this feedback including Fidler et al., and Hall et al.^17^,^18^ In my study, participants reported a remarkable improvement in their clinical skills, time management, communication skills and communication with patients.

However, some of the studies had small change in behavior in which only a few participants had responded in positive way, Sargeant et al. reported a minority of changes in his study, because they perceived feedback as negative or either inaccurate or not useful.^19^ According to few participants, the results were totally biased and that they would not accept such random judgment-based evaluation. Participants also recommended that raters should not evaluate what they could not observe

An important factor that could enhance the acceptance of MSF was effective facilitation by an appraiser, mentor or facilitator.^15^,^18^ Similar results were supported by Miller in her review of 16 studies that appropriate facilitation could lead to practice improvement.^20^

Feedback was seen to be perceived as useful only when the participants considered that the raters are well familiar with their work or they had sufficient interaction with them. According to review conducted by Burford et al. (2010) and Bracken DW and Rose DS et al. (2011), credibility of rater was identified to be an important factor. In our study, four raters were included for assessment; in literature 4 to 11 raters have been employed.^21^,^22^

In our study, participants emphasized that the feedback would be more acceptable if the raters were well-familiar with their work and have seen them in action.^19^ Some of the participant receiving negative feedback explained that doctor colleagues rarely had the opportunity to observe them in practice. Even the negative feedback was accepted more effectively if that was given by a colleague who had better rapport among the co-workers.

### Study limitations and future directions

The participants belonged to only one hospital. The study duration was six months, which was not long enough to evaluate the behavioral change. Patient’s feedback was not included in this evaluation system. Only Self-reported change was seen through focus group discussion.

## CONCLUSION

Multisource feedback is a useful tool for feedback that impacts the young doctors in many ways. It increases their sense of responsibility, management skills and self-directed learning. The improvement in individual abilities and teamwork also helped improve patient care. Factors effecting the acceptance of MSF in healthcare setting included characteristics of raters, source and format of feedback, facilitation by raters and acknowledge of deficiency.

### Authors Contribution:

**NA & AS:** Conceived and designed the study.

**NA:** Collected the data.

**NA:** Responsible and accountable for the accuracy or integrity of the work.

**AS & RAK:** Reviewed the manuscript and critically analyzed it.

All the authors were involved in analyzing the data and manuscript writing, and approved the final version.
